# A Risk-Based Approach to Evaluating Wildlife Demographics for Management in a Changing Climate: A Case Study of the Lewis’s Woodpecker

**DOI:** 10.1007/s00267-012-9953-z

**Published:** 2012-10-16

**Authors:** Erin Towler, Victoria A. Saab, Richard S. Sojda, Katherine Dickinson, Cindy L. Bruyère, Karen R. Newlon

**Affiliations:** 1 National Center for Atmospheric Research, Boulder, CO 80307 USA; 2 United States Department of Agriculture, Rocky Mountain Research Station, Bozeman, MT 59717 USA; 3 Northern Rocky Mountain Science Center, United States Geological Survey, Bozeman, MT 59715 USA; 4 Montana Natural Heritage Program, Helena, MT 59620 USA

**Keywords:** Climate change, Species vulnerability, Adaptation, Avian conservation, Forest management, Risk

## Abstract

Given the projected threat that climate change poses to biodiversity, the need for proactive response efforts is clear. However, integrating uncertain climate change information into conservation planning is challenging, and more explicit guidance is needed. To this end, this article provides a specific example of how a risk-based approach can be used to incorporate a species’ response to climate into conservation decisions. This is shown by taking advantage of species’ response (i.e., impact) models that have been developed for a well-studied bird species of conservation concern. Specifically, we examine the current and potential impact of climate on nest survival of the Lewis’s Woodpecker (*Melanerpes lewis*) in two different habitats. To address climate uncertainty, climate scenarios are developed by manipulating historical weather observations to create ensembles (i.e., multiple sequences of daily weather) that reflect historical variability and potential climate change. These ensembles allow for a probabilistic evaluation of the risk posed to Lewis’s Woodpecker nest survival and are used in two demographic analyses. First, the relative value of each habitat is compared in terms of nest survival, and second, the likelihood of exceeding a critical population threshold is examined. By embedding the analyses in a risk framework, we show how management choices can be made to be commensurate with a defined level of acceptable risk. The results can be used to inform habitat prioritization and are discussed in the context of an economic framework for evaluating trade-offs between management alternatives.

**Electronic supplementary material:**

The online version of this article (doi:10.1007/s00267-012-9953-z) contains supplementary material, which is available to authorized users.

## Introduction

Natural-resource managers are becoming increasingly concerned about the projected threat that climate change poses to ecosystems. Ominous predictions for biodiversity loss (Sala and others 2000; Thomas and others 2004) have underscored the need for proactive response efforts, but addressing climate change is challenging because of incomplete knowledge of species’ responses to climate variation and uncertainties in future climate conditions. Nevertheless, natural-resource decision makers are increasingly being asked to consider climate change in their planning [United States Department of Agriculture (USDA) 2011; United States Department of Interior (USDI) 2009], and guidance for practitioners is emerging (West and others 2009). However, most recommendations to adapt conservation to climate change have come in the form of general principles and lack the specificity needed to be “actionable” in practice (Heller and Zavaleta 2009). As such, explicit examples of how to integrate climate impacts on biodiversity into conservation planning are needed.

For resource managers, vulnerability assessments have emerged as a key tool to understand how species will respond to climate change and to inform adaptation planning (Dawson and others 2011; Glick and others 2011). As defined by the Intergovernmental Panel on Climate Change (IPCC 2007), vulnerability includes three components: sensitivity, exposure, and adaptive capacity. In terms of species vulnerability, sensitivity indicates how tolerant a species is to changing conditions, whereas exposure is the degree to which a species will experience those conditions. Adaptive capacity refers to a species’ potential to decrease their exposure or sensitivity. The traditional approach to assess climate change impacts on biodiversity has been through the use of bioclimatic envelope, or species distribution, models (Guisan and Thuiller 2005). These mainly identify exposure to climate change and provide spatially explicit shifts in species or ecosystem ranges. More recently, data, modeling, and resource limitations have motivated conservation organizations and management agencies to develop relative vulnerability indices (e.g., Bagne and others 2011; see examples in Rowland and others 2011). These question-based nonspatial assessments use a systematic evaluation framework to consider exposure, sensitivity, and adaptive capacity. The approaches are complementary because both have advantages and limitations (Rowland and others 2011), but there is a pressing need to consider all three components of vulnerability, from multiple sources, in a more integrated manner (Dawson and others 2011). To this end, conceptual vulnerability frameworks have been proposed for human–environment systems (Turner and others 2003) and specifically for species (Williams and others 2008). However, despite these conceptual advances, there is still a paucity of concrete, operational examples of adaptation principles that consider climate uncertainty (Heller and Zavaleta 2009).

Given future climate uncertainty, the use of climate scenarios has emerged as a viable tool to guide conservation (Peterson and others 2003), but how to “best” incorporate climate scenarios in adaptation planning is context dependent (Dessai and others 2005). One widespread tactic has been the “top-down” approach put forth by the IPCC, where climate scenarios are derived from downscaled projections of multiple Atmosphere Ocean General Circulation Models under different emission pathways. Subsequently, these climate scenarios are applied to impact models, such as the aforementioned species distribution models. These evaluations are useful for characterizing the consensus and range of potential impacts based on the best climate science available. However, they can be difficult to use directly in adaptation due to uncertainties in climate projections and impact models (Wilby and Dessai 2010), and they often do not address all of the factors (e.g., social, economic) or spatial scales relevant to adaptation (Burton and others 2002). An alternative is to adopt a risk-based approach (e.g., Yohe and Leichenko 2010), which has been identified as the most appropriate overarching framework for adapting to climate change (Jones and Preston 2011). Jones (2001) outlines a risk-management framework for climate impact assessments; this approach also examines impacts based on climate scenarios (although not necessarily directly derived from climate models), but it focuses more on stakeholder involvement and conveys risks relevant to decision-making by organizing analyses around the likelihood of exceeding critical thresholds. Risk management offers a systematic way to weigh likelihood and consequence, but it is also flexible in its ability to incorporate a range of approaches appropriate to different adaptation contexts (Jones and Preston 2011).

In this article, we provide a specific example of using a risk-based approach to link a species’ response to climate with conservation decisions. To demonstrate the approach, we take advantage of species’ response (i.e., impact) models that have been developed for a well-studied bird species of conservation concern. Specifically, we examine the current and potential impact of climate on nest survival of the Lewis’s Woodpecker in two different habitats. For climate scenarios, we manipulate historical weather observations to create ensembles (i.e., multiple sequences of weather events) that reflect historical variability and potential climate change. These ensembles allow for a probabilistic evaluation of the risk posed to Lewis’s nest survival. To this end, two demographic analyses are conducted: (1) the relative value of each habitat is compared in terms of nest survival; and (2) the likelihood of exceeding a critical population threshold is examined for one habitat. To support habitat management, the analyses are embedded in a risk framework where a level of acceptable risk is defined, and the climate scenarios are used to identify the climate shift that corresponds to that level. The results can be used to inform habitat prioritization and are discussed in the context of an economic framework for evaluating trade-offs between management alternatives.

## Case Study Overview

In this article, the Lewis’s Woodpecker (*Melanerpes lewis*), a well-studied bird species of conservation concern, is used to demonstrate how to link a species’ response to climate with conservation decisions. This section provides a brief background on Lewis’s Woodpecker followed by a description of previously published species’ response (i.e., impact) models that are used for the evaluation. Finally, we examine future climate projections for the case study area.

### Background

Lewis’s Woodpecker (*M. lewis*) populations are potentially decreasing at both regional and local scales (Tobalske 1997). Consequently, the species has been designated as a species of conservation concern by several state and federal agencies (Neel 1999; Ritter 2000; United States Fish and Wildlife Service 2008; USDA 2009). This woodpecker is found in open woodlands throughout the western United States, and the most productive nesting habitats are burned pine forests (Bock 1970; Raphael and White 1984; Tobalske 1997; Linder and Anderson 1998; Saab and others 2011) as well as riparian woodlands composed of cottonwoods (*Populus* spp.) (Saab and Vierling 2001) and aspens (*Populus tremuloides*) (Newlon and Saab 2011).

### Species’ Response Models

**Fig. 1:**
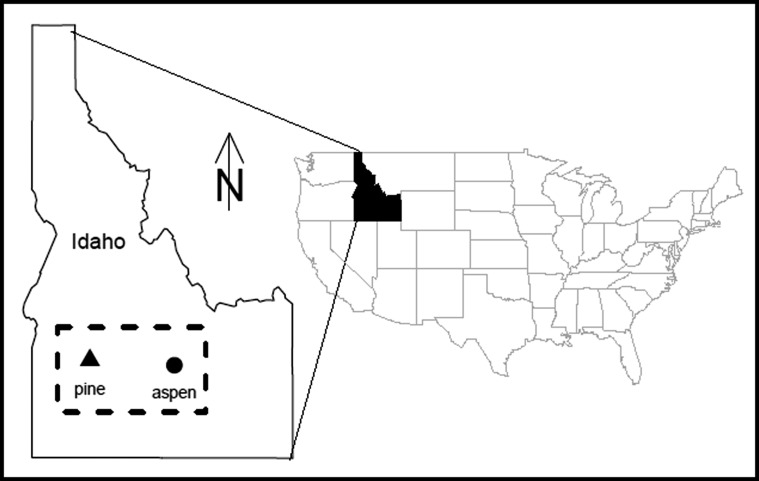
Approximate locations of nest monitoring study sites for burned pine and aspen woodlands in Idaho. Table 1 shows characteristics for each site. *Dashed box* shows approximate area used in the NRCM analysis

To understand the response of Lewis’s Woodpeckers to climate, we draw on recent efforts to characterize nest survival rates in two productive habitats: burned pine forests and aspen riparian woodlands in Idaho (Fig. 1; Table 1). For both habitats, previously published studies establish models of daily survival rate (DSR) as a logistic (logit) function. For the burned pine habitat, nests were monitored at two wildfire locations in ponderosa pine (*Pinus ponderosa*) forests of western Idaho (Boise county) (43°35′ N, 115°42′ W) during an 11 year period (from 1994 to 1999 and 2002 to 2004), representing 1 to 12 years after fire. Using data collected from these two burns, Saab and others (2011) developed the following model

**Table 1:** Characteristics of the field-monitoring studies and best-fit nest survival models

Study	Newlon and Saab (2011)	Saab et al. (2011)
Map symbol^a^	●	▲
Habitat	Aspen riparian woodlands	Burned conifer forests
Weather station (COOPID)^b^	Craters of the Moon, ID (102260)	Idaho City, ID (104442)
Field years	2002–2004	1994–2004
Sample size	76	716
Best-fit logit model:		
Intercept Cf^c^ (Cl)^d^	3.4 (0.68, 6.2)	8.8 (6.9, 10.7)
TMX Cf^c^ (Cl)^d^	0.19 (0.07, 0.31)	−0.038 (−0.060, −0.016)
PCP Cf^c^ (Cl)^d^	–	−0.21 (−0.31, −0.11)
NID Cf^c^ (Cl)^d^	−0.18 (−0.25, −0.11)	–
PFP Cf^c^ (Cl)^d^	–	−0.80 (−1.2, −0.41)

^a^Refers to Fig. 1

^b^Cooperative Station Identifier

^c^Best-fit logit model coefficient

^d^Lower and upper confidence levels

1 $$ {\text{logit}}\left( {\text{DSR}} \right) = 8.8 - 0.80\left( {\text{PFP}} \right) - 0.21\left( {\text{PCP}} \right) - 0.038({\text{TMX}}), $$

where PFP is the postfire period (=0 for the “early-burn” period of 1–4 years after fire; =1 for the “late-burn” period of 5–12 years after fire), PCP is the daily precipitation in millimeters, and TMX is the daily maximum temperature in °C. Hence, DSRs are higher in the early-burn PFP than in the late-burn and decrease with increasing PCP and TMX.

Nests were also monitored in aspen woodlands of central Idaho (Butte and Blaine counties) (43°30′ N, 113°46′ W) during three breeding seasons (2002–2004). Using data collected from this aspen location, Newlon and Saab (2011) developed the following model

2 $$ {\text{logit}}\left( {\text{DSR}} \right) = 3.4 - 0.18\left( {\text{NID}} \right) + 0.19({\text{TMX}}), $$

where NID is the nest initiation date (=1 for May 23). As such, survival rates decrease as nest initiation occurs later in the season, but they increase with higher daily TMXs.

**Fig. 2:**
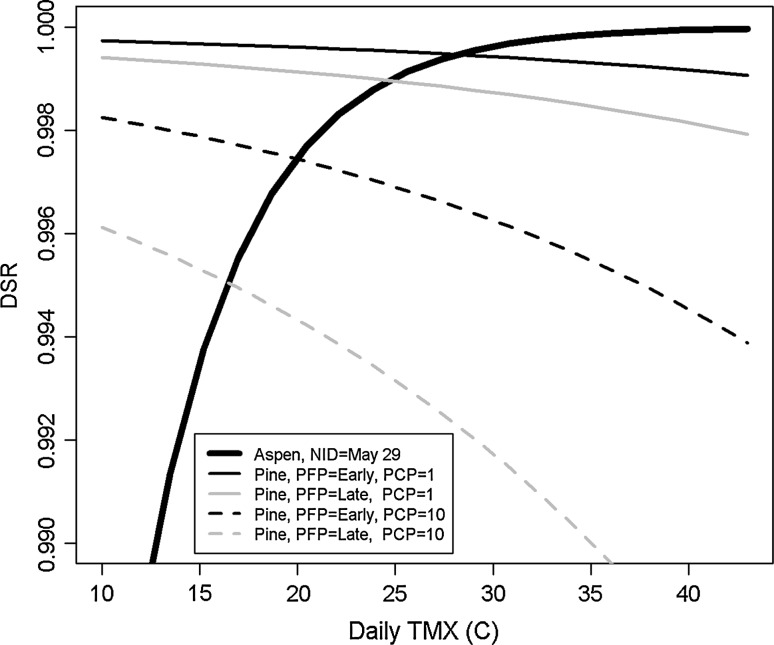
Relationship between TMX and DSR for Lewis’s Woodpeckers in aspen woodland and burned pine habitat. For aspen, NID is May 29th. For burned pine, the influence of early and late PFP, as well as varying PCP (mm), are shown

A striking difference between these two models is that increasing daily TMX results in opposite impacts: DSR increases with TMX in aspens and decreases with TMX in burned pine (Fig. 2). For burned pine, it can also be seen that increasing PCP shifts the DSR curve down as does the late-burn PFP. Here, the aspen DSR curve is shown for only one initiation date, May 29 (NID = 7); however, we note that the aspen DSR curve shifts down and right for increasing NID.

The product of each DSR value within a nesting season can be computed to obtain the nesting period or overall survival rate (OSR) for each season as follows

3 $$ {\text{OSR}} = \mathop \prod \limits_{i = 1}^{N} {\text{DSR}}_{i} , $$

where *i* is the day of the nesting season and *N* is the average number of days per nesting season.

### Climate Change Projections

The preceding response models show that Lewis’s Woodpeckers are sensitive to climate conditions, in particular, temperature and precipitation. To assess how future climate conditions might impact this species, we examined climate change projections to 2050 for the case study area. This information serves to inform our climate scenario development (see “Climate Scenarios”).

Both case study sites are located in Idaho (Fig. 1), which is part of the Pacific Northwest region (PNW). PNW projections have been extensively analyzed (Mote and Salathé 2010), but given the localized nature of this assessment, it was desirable to also focus on the study area using a new high-resolution, dynamically downscaled data set. We analyzed 36 km-resolution simulations from the National Center for Atmospheric Research (NCAR) Nested Regional Climate Model (NRCM; Holland and others 2010) embedded in the global Community Climate System Model (Collins and others 2006) for the A2 greenhouse gas (GHG) emission pathway. We compared daily NRCM simulations of current and future climate for southern Idaho (dashed box in Fig. 1) for the nesting period. Two time slices were examined: 2020–2030 to estimate 2025 and 2045–2055 to estimate 2050. The NRCM provides changes in *maximum* daily temperatures, which are required by the response models, and show an average daily TMX increase of 3.0 °C by 2050, and 1.0 °C by 2025 (figures not shown). These results are consistent with PNW multimodel results, which report average summer seasonal increases of 2.7 °C by the 2040s, ranging from 1 to 4.5 °C [estimated from Fig. 9 in Mote and Salathé (2010) for the A1B GHG emission pathway; we note that the A1B and A2 GHG emission pathways are nearly identical up to 2050].

**Fig. 3:**
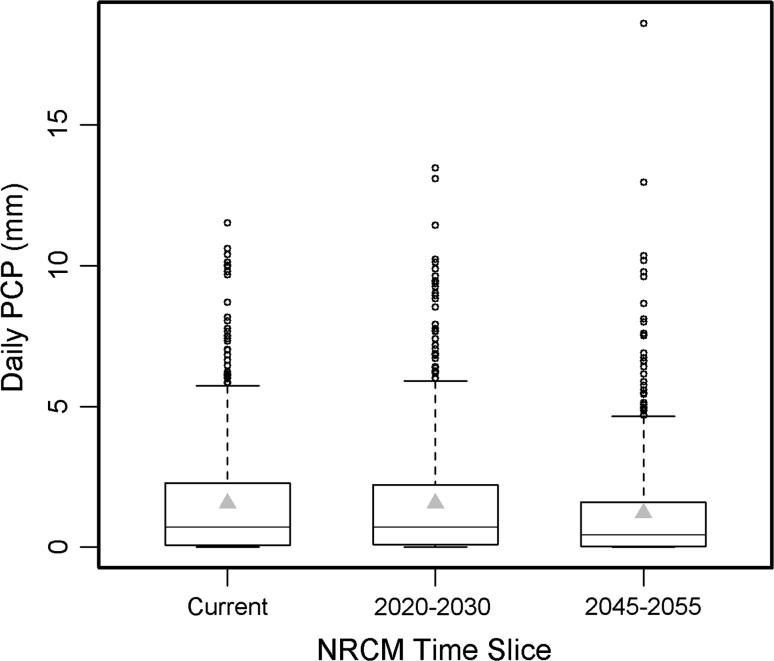
Daily PCP distribution from the NRCM projections (*box plots*) and average (*grey triangles*). Projections are raw model data (i.e., not bias-corrected)

For daily PCP, the 2025 NRCM projection for our study area showed no average change, although 2050 exhibited an average daily decrease of 23 % (Fig. 3). Modeled average precipitation (as predicted by a multimodel ensemble) displays a great deal of uncertainty (Murphy and others 2004), especially in terms of capturing summer convection (Liang and others 2004). This is underscored by the PNW multimodel analysis, which reports an average summer change of −11.2 % by 2040, but ranges from −30 to +10 % (estimated from Fig. 10 in Mote and Salathé (2010) for the A1B GHG emission pathway). However, despite this large uncertainty in average precipitation, it is generally agreed that the intensity of precipitation events is likely to increase (Meehl and others 2007). The NRCM model, being a high-resolution model, is capable of capturing such heavy localized rainfall events. It is notable that for both NRCM time slices there is a relative increase in large PCP outliers, which is consistent with the notion that precipitation extremes are likely to increase (Gutowski and others 2008; Trenberth 2011). The 2050 NRCM projection shows two values that exceed the range of current maximum daily values: increases of 13 and 61 %, respectively (Fig. 3).

## Probabilistic Risk Analysis

For the risk analysis, first we develop climate scenarios that provide a probabilistic estimate of the impact response. Second, we describe two demographic habitat assessments that use the climate scenarios in conjunction with the species’ response models.

### Climate Scenarios

Climate scenarios were developed by manipulating historical weather observations to create multiple “new” sequences of daily weather (i.e., ensembles) during the nesting season. To provide a robust characterization of climate variability as a baseline, we first create a climate scenario that reflects historical or “natural” variability. To this end, we obtained weather observations of daily TMX and PCP data from 1959 to 2009 from both weather stations (Table 1) from the National Climatic Data Center (available at: http://lwf.ncdc.noaa.gov/oa/climate/stationlocator.html). Specifically, data beginning on May 29 (NID = 7) through the *N* = 51 day nesting period (Newlon and Saab 2011) was used. We used these historical data to simulate the ensembles for the natural variability climate scenario and, subsequently, to derive the climate change scenarios.

To generate the natural variability scenario, we adopt a simple daily disaggregation technique based on resampling historical proportional vectors (Nowak and others 2010). The stochastic disaggregation approach has been well tested for streamflow (Nowak and others 2010; Towler 2010), and the step-by-step approach is detailed in Online Reference 1. In short, the natural variability climate scenario is comprised of 250 ensembles, where each ensemble is comprised of PCP and TMX for the 51 day nesting season. The approach faithfully preserved the historical distributional statistics for PCP and TMX at the seasonal and daily timescales; sample validation results are shown in Online Reference 2.

Next, climate change scenarios are developed. We derive two independent scenario sets from the natural variability scenario that were relevant to each of the forthcoming assessments: the delta TMX and the above-PCP scenarios.

#### Delta TMX Scenarios

**Fig. 4:**
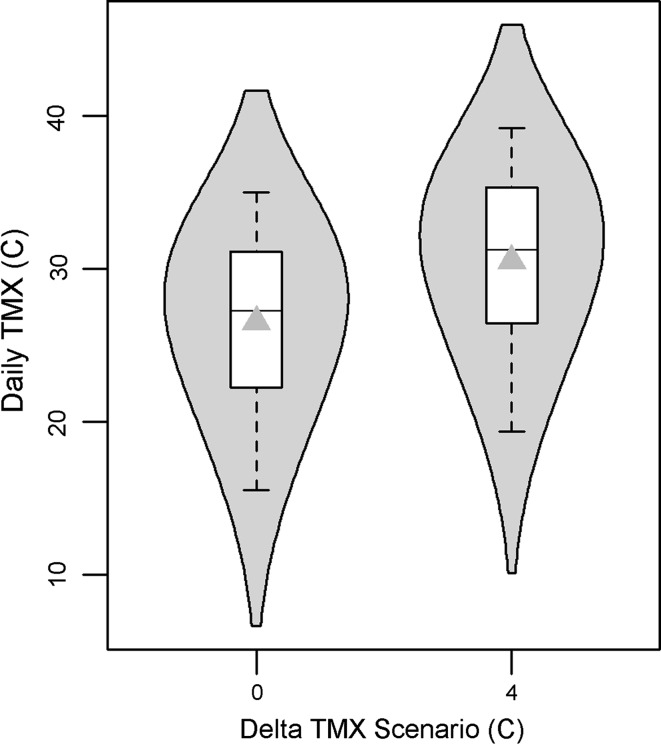
Daily TMX distribution for simulated nesting seasons (*box plot*) and simulation average (*grey triangles*) for the aspen woodland study area. Kernel-density plot (*grey* “*clouds*”) is overlaid

To reflect the increasing temperatures projected by the climate models (see “Climate Change Projections”), a simple delta method was applied to the daily TMX values of the natural variability scenario. Ensembles reflecting a delta TMX from 0 (i.e., the natural variability scenario) to 10 °C were developed. Here, because of the translational change applied, the distribution maintains its shape but shifts higher by the delta (Fig. 4). In terms of weather variables, we note that the aspen response model only includes TMX, but the burned pine model includes both TMX and PCP. As such, for the burned pine model, the daily PCP sequences remain unchanged (i.e., the natural variability scenario) for each of the delta TMX scenarios. Although increasing temperatures are inversely correlated with PCP, this was negligible here because the majority of the burned pine survival rate distribution was more sensitive to TMX than to PCP. Hence, this technique was useful for comparing the OSR distributions between aspen and burned pine habitats (i.e., in the forthcoming “Comparison of Alternatives” assessment). However, the lower tail of the burned pine distribution was sensitive to high values of PCP, thus motivating the development of the above-PCP scenarios, described in the next section.

#### Above-PCP Scenarios

Although modeled precipitation response remains uncertain, extremes are likely to increase (see “Climate Change Projections”). Through 2050, natural variability is likely to play a dominant role, especially at smaller spatial and temporal scales (Hawkins and Sutton 2009). Given these findings, natural variability and extreme values are the most important considerations for PCP.

**Fig. 5:**
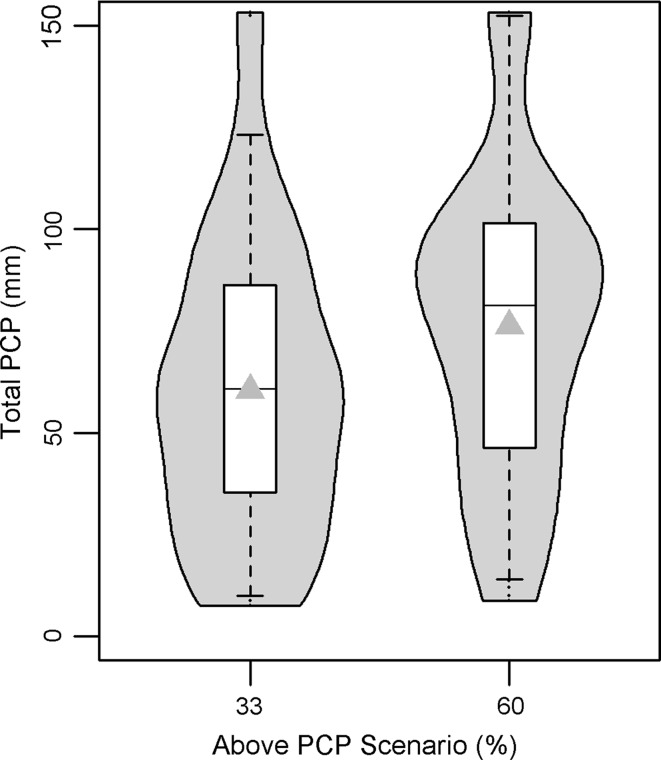
Total PCP distribution for simulated nesting seasons (*box plot*) and simulation average (*grey triangles*) for the burned pine study area. Kernel-density plot (*grey* “*clouds*”) is overlaid

To contend with these issues, precipitation scenarios were developed using a tercile approach based on the format used by climate-forecasting agencies, such as the National Oceanic and Atmospheric Administration (NOAA). The advantage is a seamless framework that can span seasonal to decadal time scales. Specifically, the *A*:*N*:*B* format is used, which indicates the probability that the season will fall within the “above,” “normal,” or “below” historical tercile. Hence, *A*:*N*:*B* = 33:33:33 represents the natural variability scenario. The PCP scenarios were developed by shifting the probability of resampling the above-PCP tercile: This was performed from 0 to 100 %, in steps of 10 %, while keeping the normal-PCP and below-PCP tercile probabilities equal. For instance, when *A* = 60, *B* = *N* = 20 and so on. Figure 5 shows the distributional changes for seasonal precipitation for the 33 % (natural variability) and the 60 % above-PCP scenarios. The distributional range is the same for both scenarios, but for the 60 % scenario, more of the values are resampled from the upper part of the distribution, thus causing the kernel density distribution (grey cloud) to bulge just <100 mm. This technique is justified by the fact that seasons with high total rainfall also tend to contain larger rainfall events, thus the frequency of upper “extreme” PCP values increases with higher above-PCP scenarios. Compared with the delta TMX scenarios, these scenarios were tailored for an assessment of burned pine that hinged on the OSR distribution tail [i.e., the threshold exceedance likelihood assessment (see “Threshold Exceedance Likelihood”)].

### Demographic Assessment by Habitat

#### Comparison of Alternatives

The delta TMX scenarios were used to compare the current and potential impact of climate on nest survival of the Lewis’s Woodpecker in two different habitats. In other words: Is aspen or burned pine habitat more valuable in terms of nest survival? For decision making, this information is useful for prioritization of habitat conservation and restoration. For simplicity, we consider only the survival of Lewis’s Woodpecker, as measured by OSR, in valuing these habitats.

The OSR distributions of each habitat are compared using a nonparametric significance test. The more the distributions overlap, the more similarly the habitats are valued. Conversely, as the OSR distributions diverge, one habitat gains import over the other [assuming constant habitat quality (see discussion in “Strengths and Limitations”)]. The underlying concept of this approach is the basis for many well-known parametric significance tests (e.g., Student *t* test), but the nonparametric approach is appealing for its lack of assumptions, intuitive nature, and ability to be paired with stochastic simulations. The approach does require identifying a subjective confidence level; here we examine three cases: 50 % (case A), 75 % (case B), and 95 % (case C). Case A is the tipping point, where at least 50 % of one distribution (i.e., the median) is above the other. Case B, a moderate decision threshold, is achieved when at least 75 % of the distribution is offset (i.e., the 75th percentile of one is above the 25th percentile of the other), and so on for the more stringent case C. Although subjective, the confidence level can be chosen by a decision maker to be commensurate with the risk she or he is willing to take. Alternatively, the overlap fraction can be read directly and used to set decision weights. For example, case B could justify allocating 75 % (25 %) of available resources to projects that protect/improve the better (worse) habitat.

#### Threshold Exceedance Likelihood

The above-PCP scenarios were used to evaluate the likelihood of exceeding a critical threshold, which can serve as relevant organizing points for quantifying risk (Jones 2001). Here, we focus on the early-burn pine and examine the likelihood that the habitat will be a population sink for the Lewis’s Woodpecker, that is, that the habitat will not be adding an increasing number of recruits to the overall population. Calculated from Saab and Vierling (2001), early-burn pine is a sink if the OSR value is <0.49. From the OSR distribution, we can directly obtain the percent of data below the sink threshold, thus resulting in a measure of the associated risk. Unfortunately, this type of threshold information was not available for the late-burn pine or aspen habitats for comparison.

In this case, whether or not the early-burn pine habitat is a population sink is primarily sensitive to extreme PCP events; thus, the above-PCP scenarios are appropriate. Other demographic processes (e.g., predation) that potentially affect source–sink relationships were not considered here.

## Results

**Fig. 6:**
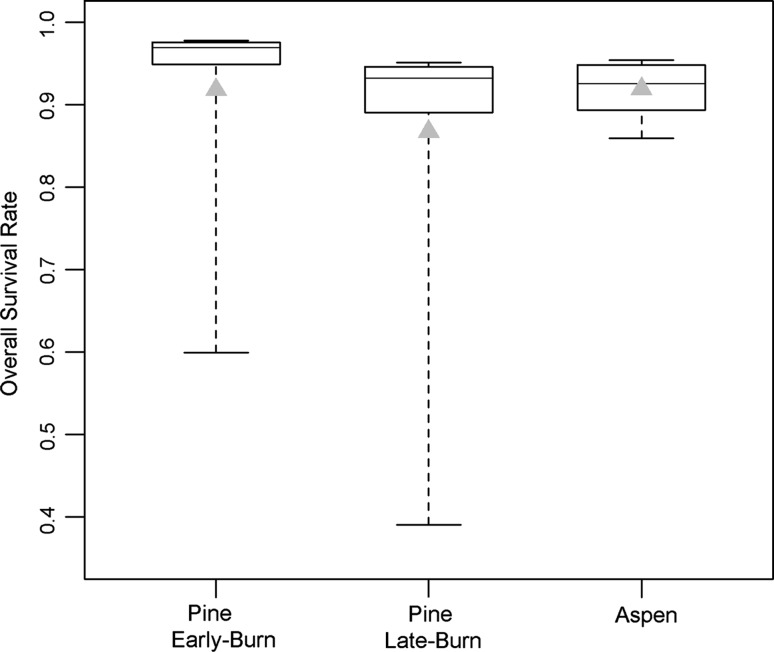
Overall survival rate of Lewis’s Woodpecker for natural variability scenario in burned pine and aspen habitats for simulated nesting seasons (*box plots*) and simulation average (*grey triangles*)

Using the delta TMX scenarios, aspen and burned pine habitat were compared. To start, natural variability for each habitat was characterized (Fig. 6), illustrating that early-burn pine has the highest survival rate (median = 0.969). The natural variability distributions show that there is at least 75 % confidence that the early-burn pine OSR values are greater then the other two habitat types, thus satisfying cases A and B. Late-burn pine has a slightly higher median OSR (0.932) than aspens (0.924), thus satisfying case A, although the bulk of the distributions are similar in range. One notable difference is that for both burned pine habitats, the distribution is skewed toward lower values as evident by both (1) the mean being below the interquartile range and (2) the extent of the bottom whisker; this point will be discussed later in this section.

**Fig. 7:**
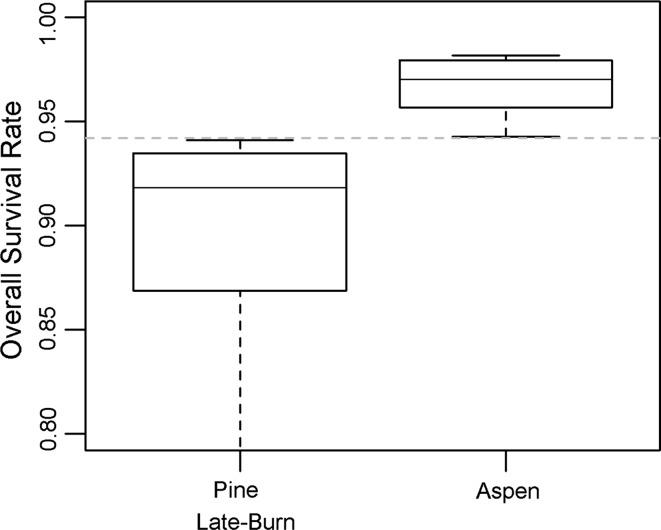
Overall survival rate for an increase in daily TMX of 5 °C (delta TMX = 5 scenario) shown for late-burn pine and aspen habitats. *Horizontal dashed grey line* is the risk threshold for which 95 % of the data does not overlap

Next, the results for the increasing delta TMXs were examined. To focus on results that are relevant for a risk-based decision approach, we identify the climate scenarios for which each predefined case is achieved (Table 2). For a 1 °C shift, the aspen habitat median increases to above the late-burn pine (case A). For a 4 °C shift, aspens satisfies case B (case A) compared with late-burn pine (early-burn pine). Figure 7 shows that case C is achieved for aspens versus late-burn pine for delta TMX = 5. It takes 9 °C to satisfy case C when comparing aspens and early-burn pine (Table 2). Thus, the overall pattern is that as temperature increases, aspen habitat becomes increasingly competitive with the burned habitats, especially with late-burn pine. Again, these results assume that the habitat quality is constant under shifting climate (see discussion in “Strengths and Limitations”).

**Table 2:** Daily TMX increase that would achieve each risk-based confidence level (i.e., case) for overall survival rate

	Delta TMX (C)	
	Aspen versus pine (early-burn)	Aspen versus pine (late-burn)
Case A (50th and 50th tie)	4	1
Case B (25th and 75th tie)	7	4
Case C (5th and 95th tie)	9	5

**Fig. 8:**
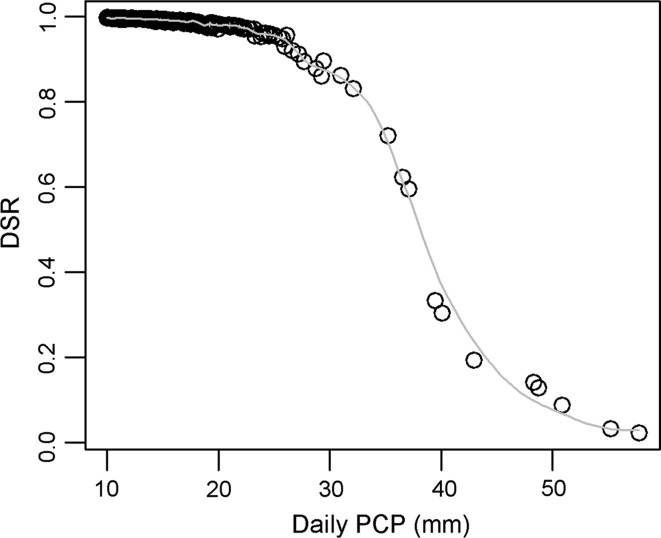
Simulated daily PCP values (>10 mm) and corresponding DSR values in early-burn pine habitat. *Gray line* is local smoother

As mentioned, the OSRs of the burned pine habitat exhibit a strong negative skew because the average is below the interquartile range (Fig. 6). This is caused by low survival rate outliers, which are being driven by high PCP (Fig. 8). As daily PCP increases >25 mm, there is a dramatic decrease in DSR. This relationship illustrates the classic paradigm of a low-probability, high-consequence event.

**Fig. 9:**
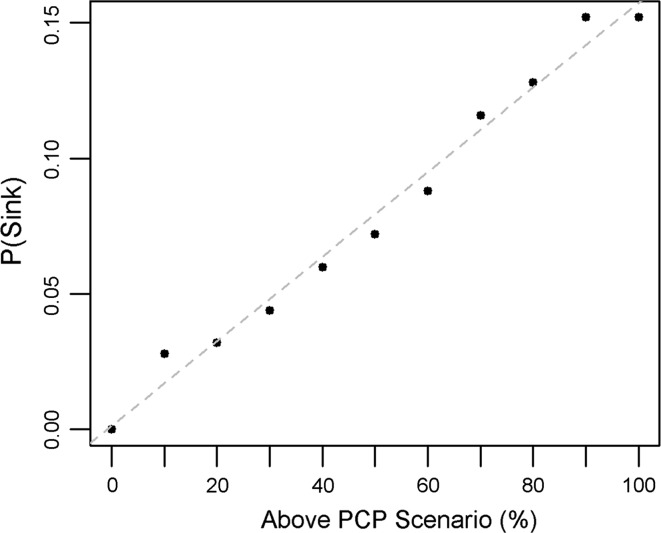
In early-burn pine habitat, the risk of a season being a population sink [P(Sink)] for above-PCP scenarios. *Grey dashed* is best linear fit (*y* = 0.16*x*)

The threshold exceedance analysis illustrates this risk, which is focused on the sink threshold of OSR <0.49. For early-burn pine, this was examined for each of the above-PCP scenarios, showing the increase in sink likelihood in a given season (Fig. 9). In the natural variability scenario, where the above-average precipitation chance is 33 %, the likelihood of early-burn pine being a sink is approximately 5.3 %. Furthermore, for every 10 % increase in the chance of being in the above-PCP category, there is a 1.6 % risk increase. For the 60 % above-PCP scenario, the risk is approximately 9.5 %, and the range spans from 0 to 16 % chance (for the 0 to 100 % scenarios, respectively).

## Discussion

### Decision Application

Given that the purpose of this study was to integrate climate impacts on biodiversity into decision-making, the next step is to explore how the results from the demographic analyses can be incorporated into conservation planning. We begin by considering the results listed in Table 2 in light of the climate change projections examined in “Climate Change Projections”. First, we consider a comparison of aspens with early-burn pine (i.e., the first 4 years after a burn). Although increasing temperatures are associated with increased nest survival in aspen habitat, if we use the NRCM 2050 projection of 3 °C as a guide, then early-burn pine is likely to remain more valuable in terms of nest survival than aspen habitat because aspen does not satisfy case A until 4 °C (Table 2). One caveat is that Lewis’s Woodpecker nesting in early-burn pines is vulnerable to high (extreme) precipitation events, thus the habitat’s value is decreased under wetter precipitation outlooks. For example, if one accepts a population sink risk ≤10 %, then the early-burn pine remains the top priority habitat unless the above-PCP forecast is >60 %. Barring that scenario, it seems that the early-burn habitat’s dominance will be robust to temperature increases projected up to 2050. Subsequently, the evaluation between aspens and late-burn pine (i.e., 5 to 12 years after a fire) comes into play. Under natural variability, late-burn pine holds a slight advantage, although its lead is tenuous using the NRCM 2025 projection of 1 °C as a guide (Table 2). Aspen achieves the moderate and stringent cases with 4 °C and 5 °C, respectively, which is approximately the upper range of the regional multimodel projections. Although other factors must be considered, our results provide managers with quantitative information that could be integrated into an adaptive-management design and can help managers place their objectives, actions, and evaluations into a long-term perspective.

To illustrate this utility, we describe a hypothetical decision protocol to allow salvage logging in a postburn forest. From an economic perspective, there may be an opportunity cost to forgoing logging revenue that could be put to alternative uses. Although carefully designed salvage logging can provide suitable nesting habitat for Lewis’s Woodpecker (Saab and others 2007), clear-cutting removes all nesting habitat. For the purpose of this exercise, we propose a simple analysis between two choices: (1) allow clear-cutting in burned pine and use the funds to restore aspen (i.e., directly invest in aspen) or (2) do not allow clear-cutting (i.e., indirectly “invest” in burned pine habitat). In this example, the equation we are seeking to maximize is expressed as follows

4 $$ V = V_{\text{Act}} + V_{\text{Bird}} , $$

where *V* is the total value in dollars, *V*_Act_ is the cost/revenue associated with the choice, and *V*_Bird_ is the value of the benefit attributed to the survival of Lewis’s Woodpecker in the area under consideration. Although the latter is difficult to quantify, economists have developed a range of techniques to measure the value of nonmarket goods and services (e.g., Champ and others 2003). These values might include the “existence value” individuals attach to knowing that the Lewis’s Woodpecker is thriving as well as recreational values (e.g., bird watching tours). For choice 1, Eq. 4 becomes the following

5 $$ V_{1} = \left( {L - R} \right) + W(OSR_{\text{Aspen}} ), $$

where *L* is the logging revenue, *R* is the aspen restoration cost, and W is the dollar value assigned to the woodpecker’s survival. We assume that *W* > 0 or that there is some positive value attached to bird survival. For choice 2, there is no action, and therefore no cost/revenue generated, as follows

6 $$ V_{2} = 0 + W({\text{OSR}}_{\text{Pine}} ). $$

In this simplified set-up, logging (choice 1) will be chosen when *V*_1_ is >*V*_2_:

7 $$ \left( {L - R} \right) + W\left( {{\text{OSR}}_{\text{Aspen}} } \right) > W\left( {{\text{OSR}}_{\text{Pine}} } \right). $$

Or, solving in terms of knowns (OSRs) and unknowns (*L*, *R*, *W*)

8 $$ \frac{(L - R)}{W} > ({\text{OSR}}_{\text{Pine}} ) - \left( {{\text{OSR}}_{\text{Aspen}} } \right). $$

A matrix illustrates how each of the variables influences the decision (Table 3). For instance, when (*L*−*R*) is positive (i.e., logging generates enough revenue to cover all of the restoration expenses) and aspens are a more valuable habitat than burned pine, then there is no trade-off between the revenue (*V*_Act_) and the ecological (*V*_Bird_) outcomes, thus favoring choice 1 (top-left quadrant). Similarly, when (*L*−*R*) is negative and OSR_Pine_ minus OSR_Aspen_ is positive, Eq. 8 is never satisfied, thus favoring choice 2 (bottom-right quadrant).

**Table 3:** Decision matrix between two choices: (1) allow clear-cutting in burned pine and use the funds to restore aspen or (2) do not allow clear-cutting

	OSR_Pine_−OSR_Aspen_^a^	
(*L*−*R*)^b^	− (Aspen)	+ (Pine)
+	Favors choice (1)	Depends^c^
−	Depends^c^	Favors choice (2)

See Eq. 8 and text for details

^a^OSR difference between burned pine and aspen

^b^Logging revenue (*L*) minus the cost of aspen restoration (*R*)

^c^See text for conditions that favor each choice

In the other cases, both sides of Eq. 8 have the same sign (positive or negative), thus indicating that parameter magnitudes become important. Here, the revenue (*V*_Act_) and ecological criteria (*V*_Bird_) are in conflict. When logging generates net revenues, but burned pine provide a more valuable habitat (top-right quadrant), Eq. 8 will be satisfied (not satisfied) and favor choice 1 (2) when:

- the value of *W* is small (large);
- net revenues from logging, *L*−*R*, are large (small); and/or
- the survival rate advantage of burned pine over aspens is small (large).

Meanwhile, in the bottom-left quadrat of Table 3, logging to restore is net costly but allows investment in the higher-survival aspen habitat. In this case, Eq. 8 is more likely to be satisfied and favor choice 1 when:

- the value of *W* is large;
- net losses, *L*−*R*, are small (i.e., less negative); and/or
- the survival rate advantage of aspens compared with burned pine is large (i.e., right-hand side is more negative).

Note that the decision matrix includes a criterion to calculate a difference between OSR values. Because of the stochastic approach taken here, whether that difference is positive or negative can be determined by its achievement of the aforementioned confidence levels (i.e., cases A, B, and C).

We present this example to illustrate how climate information can be applied to wildlife demographic data and used to inform management decisions. An actual land-management application likely would be more complex than the example outlined here. In the real world, input from stakeholders would be critical to define the relevant set of options and, most likely, more than two alternatives must be considered. We also acknowledge that the type of environmental valuation alluded to here, i.e., attaching a dollar value to the survival of Lewis’s woodpeckers in a particular region, is both technically difficult and perhaps philosophically objectionable to some. However, land-management alternatives inherently involve trade-offs across competing objectives. Careful attempts to make trade-offs explicit enhance transparency and force a more frank discussion of how to consider trade-offs under different contexts.

### Strengths and Limitations

Although it is not meant to be a comprehensive comparison between all Lewis’s Woodpecker habitats, our framework provides an important step toward bridging the gap between climate impacts and management actions. However, because the analysis aims to inform decision making, the results must be reviewed with awareness of the associated limitations. Here, we discuss how this study handles two key concepts that have impeded previous adaptation planning efforts: (1) uncertainties in future climate conditions and (2) how species will respond to climate variation.

To address uncertainties in future climate conditions, we used climate scenarios. First, emphasis was placed on characterizing natural variability, which was subsequently used to develop the climate change scenarios. The climate change scenarios were developed to be relevant to the demographic assessments and informed by climate model projections. Nevertheless, using climate scenarios to identify the climate shifts that are relevant to risk-based decision making (i.e., Table 2) insured that we were not beholden to any one climate model; thus, the analysis would continue to be relevant with updated projections. As shown in this article, managers can examine how the identified climate scenarios compare with projections from high-resolution downscaling efforts and coarser multimodel consensus and ranges (also see Mote and others 2011 for discussion of selecting and combining climate model projections).

To understand how Lewis’s Woodpecker responds to climate variation, we used previously developed nest survival models that included weather predictors. We recognize that adequate response data are often lacking to explore these types of quantitative relationships; nevertheless, when they do exist, we encourage their use. If they do not exist, but climate is suspected to be an important factor, then monitoring projects may be needed to develop relevant data sets. When this is not possible, managers may need to rely more on complementary relative vulnerability approaches (e.g., Bagne and others 2011; also see Rowland and others 2011).

We also note that in terms of the impact (i.e., nest survival) models, two types of uncertainty, parameter and structural, were not explicitly considered. Characterizing parameter uncertainty would be relatively straightforward through techniques such as Monte Carlo simulation. For example, the parameter distribution could be constructed using the upper and lower confidence limits included in Table 1. The structural (or model) uncertainty of the impact models is a more difficult issue. We only examine one “best-fit” model for each habitat, but it is possible that other predictors might be relevant under conditions not observed during the field campaign. Furthermore, the impact models only address one component of population dynamics: the survival rate. These results could be embedded in a more comprehensive population dynamics model.

Related to these points, we reiterate that the impact models used do not explicitly consider potential nonstationarities. For example, these results assumed that habitat quality would remain constant over time. However, projections by Rehfeldt and others (2009) suggest that the area occupied by aspen could decrease rapidly during the course of the 21st century. Attempts to model this would add additional complexity, but promising approaches include landscape-based models (e.g., Turner and others 2008) and statistically derived methods, such as hierarchical Bayesian modeling (Gelman 2004).

## Conclusion

This study provides a concrete example of how to use a risk-based approach to inform species and habitat management. Furthermore, this is the only study to date that explores the impact of climate change on the Lewis’s Woodpecker. Consistent with the individual species–based approach that typically guides conservation efforts (Glick and others 2011), this framework was showed for the Lewis’s Woodpecker. However, the approach is general and could be readily extended to other species where adequate response variable data exist. Of course, conservation efforts should draw from multiple sources and approaches (Dawson and others 2011), and we offer this as a contribution toward that goal.

## Electronic supplementary material

Below is the link to the electronic supplementary material.

Supplementary material 1 (DOCX 54 kb)

Supplementary material 1 (DOCX 86 kb)
