# Supplementary material for: A Risk-Based Approach to Evaluating Wildlife Demographics for Management in a Changing Climate: A Case Study of the Lewis’s Woodpecker
Source: Environ Manage. 2012 Oct 16;50(6):1152–63. doi: 10.1007/s00267-012-9953-z (PMC3497959; doi:10.1007/s00267-012-9953-z)
Supplement: Supplementary file 1 — Supplementary material 1 (DOCX 54 kb) [file 267_2012_9953_MOESM1_ESM.docx]

Online Reference 1

Title: A risk-based approach to evaluating wildlife demographics for management in a changing climate: A case study of the Lewis’s Woodpecker

Journal: Environmental Management

Authors: Erin Towler, Victoria A. Saab, Richard S. Sojda, Katherine Dickinson, Cindy L. Bruyère, and Karen R. Newlon

Corresponding Author: Erin Towler, National Center for Atmospheric Research (NCAR), Boulder, CO, towler@ucar.edu.

Methodology for simulating the natural variability climate scenario

The first climate scenario that is simulated is the natural variability climate scenario. For each station (i.e., Crater’s of the Moon, ID for the aspen woodlands study site, and Idaho City, ID for the burned pine study site), historical observations of PCP and TMX (see Section 2.3 in the manuscript) are used to create ensembles. These ensembles are “new” sequences of weather events, but preserve the historical climate statistics. To generate these ensembles, we adopt a simple simulation technique that has two parts: (1) seasonal resampling (Figure S1) and (2) daily disaggregation (Figure S2). Each part is briefly summarized, below:

*Part1. Seasonal resampling*

To characterize the full range of natural variability, the seasonal records (i.e., from 1959 to 2009) are bootstrapped (Efron and Tibshirani 1993) with replacement. First, a vector of the total PCP, or the sum of daily PCP from each historic 51-day nesting season, is constructed (i.e., 51 years of total PCP values). Next, the total PCP records are sorted and divided into terciles. We adopt this tercile-based approach because climate forecasting centers, such as the National Oceanic and Atmospheric Administration (NOAA) and the International Research Institute (IRI), provide their seasonal forecasts this way. The advantage is a seamless framework that can span seasonal to decadal time scales. Specifically, they use the format of “A:N:B”, which indicates the probability that the season will fall within the “above-”, “normal-”, and “below-” tercile. Hence, natural variability is characterized by a resample of A:N:B=33:33:33. As such, in the seasonal resampling for the natural variability climate scenario, 33% of the resample was taken from the above-tercile, 33% was from the normal-tercile, and 33% was from the below-tercile. Resampling was performed to create a simulation, Z, of 250 total PCP members (Figure S1).

*Part 2. Daily disaggregation*

Because the response models require daily time step values, the simulated vector of total PCP values, Z, needs to be dissagregated. In water resources, many efforts have aimed to dissaggregate streamflow (Grygier and Stedinger 1988, Stedinger and Vogel 1984, Valencia and Schaake 1973, Prairie et al. 2007, Tarboton et al. 1998), but few have been able to effectively reproduce daily time scales. Recently, Nowak et al. (2010) developed a simple method based on resampling historical proportion vectors. The technique has been succesfully utilized in streamflow-related drinking water applications (Towler et al. 2012), and here it is readily extended to a seasonal precipitation dissagregation. The reader is referred to Nowak et al. (2010) for additional details, but the main steps are provided here in brief:

1. For each season of the historic record, the observed daily precipitation values, *w*, are converted to a proportion of the season’s total PCP. The resulting fractional matrix *P*, will have dimensions 51 x 51 (i.e., years by days).
2. For each simulated total PCP, *Z*, the “nearest neighbors” are identified (i.e., the most similar historical seasons in terms of total PCP) and one of them (say, year y) is selected using a weighted probability metric (see Lall and Sharma 1996). The corresponding proportion vector (p_y_) is applied to the simulated value to obtain the daily PCP vector (z_y_), such that:

$$z_{y}=p_{y}Z$$

In short, the simulated total PCPs (Z) are scaled by a proportion

vector (p_y_), thus generating “new” daily PCP sequences (z_y_) (Figure S2a).

1. For the selected year, y, the TMX vector for that year, TMX_y_, becomes part of the simulation ensemble. As such, for a given simulation, the daily TMX sequences remain the same, but they are paired with different daily PCP sequences (z_y_) (Figure S2a).
2. Repeat steps (ii) and (iii) for all the simulations for (i) (i.e., all 250 simulations). This generates paired ensembles of daily PCP and TMX (Figure S2b).

In summary, the natural variability climate scenario is comprised of 250 ensembles of PCP and TMX for the 51-day nesting season (Figure S2b). See Online Reference 2 for validation of the natural variability climate scenario.

Figure S1. Overview of Step 1 of the disaggregation procedure.

Figure S2. Overview of Step 2 (ii)-(iii) (a) and Step 2 (iv) (b) of the disaggregation procedure.

References

Efron B, Tibshirani R (1993) An Introduction to the Bootstrap. Chapman & Hall, New York.

Grygier JC, Stedinger JR (1988) Condensed disaggregation procedures and conservation corrections for stochastic hydrology. *Water Resour Res* 24(10): 1574-1584.

Lall U, Sharma A (1996) A nearest neighbor bootstrap for resampling hydrologic time series. *Water Resour Res* 32(3): 679-693.

Nowak K, Prairie J, Rajagopalan B, Lall U (2010) A nonparametric stochastic approach for multisite disaggregation of annual to daily streamflow. Water Resour Res 46: W08529. doi:10.1029/2009WR008530.

Prairie J, Rajagopalan B, Lall U, Fulp T (2007) A stochastic nonparametric technique for space-time disaggregation of streamflows. *Water Resour Res* 43(3): W03432.

Stedinger JR, Vogel RM (1984) Disaggregation procedures for generating serially correlated flow vectors. *Water Resour Res* 20(1): 47-56.

Tarboton DG, Sharma A, Lall U (1998) Disaggregation procedures for stochastic hydrology based on nonparametric density estimation. *Water Resour Res* 34(1): 107-119.

Towler E, Raucher B, Rajagopalan B, Rodriguez A, Yates D, Summers RS (2012) Incorporating climate uncertainty in a cost assessment for a new municipal source water. *Journal of Water Resources Planning and Management-Asce,* (accepted).

Valencia D, Schaake JC (1973) Disaggregation processes in stochastic hydrology. *Water Resour Res* 9(3): 580-585.
