# Supplementary material for: A Risk-Based Approach to Evaluating Wildlife Demographics for Management in a Changing Climate: A Case Study of the Lewis’s Woodpecker
Source: Environ Manage. 2012 Oct 16;50(6):1152–63. doi: 10.1007/s00267-012-9953-z (PMC3497959; doi:10.1007/s00267-012-9953-z)
Supplement: Supplementary file 2 — Supplementary material 1 (DOCX 86 kb) [file 267_2012_9953_MOESM2_ESM.docx]

Online Reference 2

Title: A risk-based approach to evaluating wildlife demographics for management in a changing climate: A case study of the Lewis’s Woodpecker

Journal: Environmental Management

Authors: Erin Towler, Victoria A. Saab, Richard S. Sojda, Katherine Dickinson, Cindy L. Bruyère, and Karen R. Newlon

Corresponding Author: Erin Towler, National Center for Atmospheric Research (NCAR), Boulder, CO, towler@ucar.edu.

Validation of the natural variability climate scenario

The stochastic disaggregation approach has been well tested for streamflow (Nowak et al. 2010; Towler et al. 2012), but a sampling from the method validation to disaggregate seasonal precipitation is provided here. The simulation technique was used to generate ensembles of PCP and TMX, from which a suite of distributional statistics at the seasonal and daily time scales was validated. The simulation methodology can be found in Online Reference 1.

The probability density function (PDF) of the historical and simulated total nesting season precipitation is shown in Figure S1, and it can be seen that the bimodal historical PDF is very well described by the simulations. As this would indicate, all of the seasonal distributional statistics (such as mean, variance, skew, etc.) are also well reproduced (figures not shown).

Given that the nest survival models require daily values as inputs, validating the characteristics of the daily PCP simulations is also of great importance. In terms of extremes, the maximum PCP value per simulated season was examined (Figure S2, left), which is important due to its negative effect on survival rates. Similarly, it is important that the algorithm captures days without rainfall (Figure S2, right). In addition, the algorithm is able to reproduce the day-to-day sequencing that is critical for biological systems and for calculating realistic OSRs. To this end, several correlations were verified, including the lag-1 correlation for daily PCP, as well as the daily PCP to TMX correlation (i.e., lag-0; Figure S3). As we would expect, there is a positive lag-1 correlation between days with precipitation, and a negative correlation between rainfall and TMX (i.e., TMX is lower when it rains). The algorithm also captures the PDF and correlations for daily TMX (figures not shown).

*
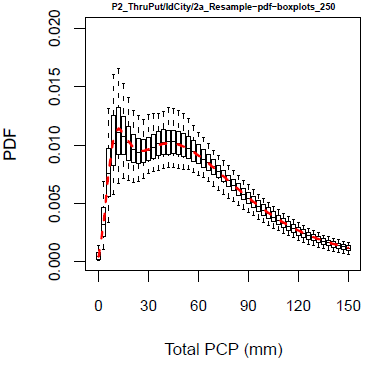
*

Figure S1. For the burned pine study site, the probability density function (PDF) of the nesting season total precipitation (PCP) simulations (box plots) and average historic (red dashed line).

*
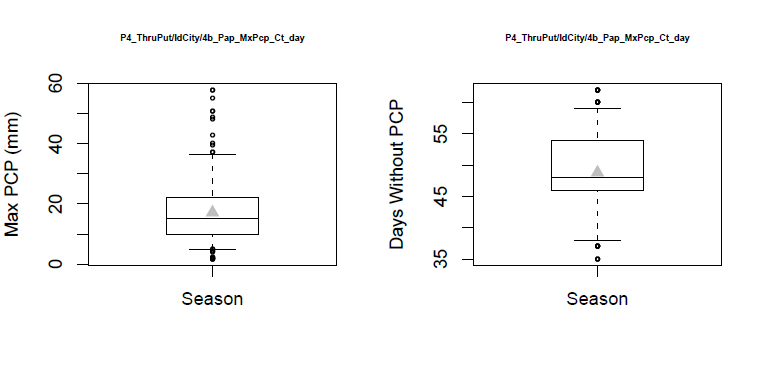
*

Figure S2. For the burned pine study site, daily maximum precipitation (PCP; left) and days without precipitation (right) for each simulated nesting season (box plot) and average historic (grey triangle).

*
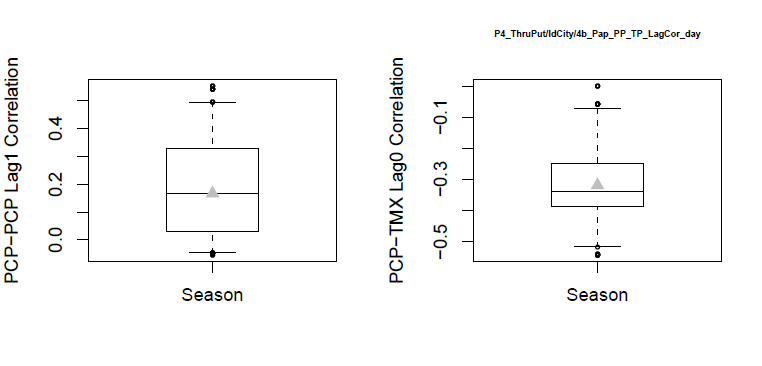
*

Figure S3. For the burned pine study site, daily precipitation (PCP) lag-1 correlation (left) and daily precipitation-maximum temperature (TMX) lag-0 correlation (right) for all simulated nesting seasons (box plot) and historic average (grey triangle).

References

Nowak K, Prairie J, Rajagopalan B, Lall U (2010) A nonparametric stochastic approach for multisite disaggregation of annual to daily streamflow. Water Resour Res 46: W08529. doi:10.1029/2009WR008530.

Towler E, Rajagopalan B, Yates D, Rodriguez A, Summers RS (2012) An integrated approach to simulate the impacts of climate change on stream water quality for municipal water use. Water Resources Management (in review).
